# Molecularly Imprinted Polymer Nanoparticles for Formaldehyde Sensing with QCM

**DOI:** 10.3390/s16071011

**Published:** 2016-06-30

**Authors:** Munawar Hussain, Kira Kotova, Peter A. Lieberzeit

**Affiliations:** Faculty for Chemistry, Department of Physical Chemistry, University of Vienna, Währinger Strasse 42, A-1090 Vienna, Austria; munawar_arif@hotmail.com (M.H.); Kira.Kotova@univie.ac.at (K.K.)

**Keywords:** molecularly imprinted polymer, quartz crystal microbalance, formaldehyde detection, MIP nanoparticles

## Abstract

Herein, we report on molecularly imprinted polymers (MIPs) for detecting formaldehyde vapors in air streams. A copolymer thin film consisting of styrene, methacrylic acid, and ethylene glycol dimethacrylate on quartz crystal microbalance (QCM) yielded a detection limit of 500 ppb formaldehyde in dry air. Surprisingly, these MIPs showed specific behavior when tested against a range of volatile organic compounds (VOCs), such as acetaldehyde, methanol, formic acid, and dichloromethane. Despite thus being a suitable receptor in principle, the MIPs were not useful for measurements at 50% humidity due to surface saturation by water. This was overcome by introducing primary amino groups into the polymer via allyl amine and by changing the coating morphology from thin film to nanoparticles. This led to the same limit of detection (500 ppb) and selectivity as before, but at the real-life conditions of 50% relative humidity.

## 1. Introduction

Formaldehyde, a pungent-smelling gas, is regarded one of the main toxic indoor pollutants [1,2,3] and increasingly draws attention to itself also in outdoor environments [4]. It is carcinogenic and causes a range of conditions including central nervous system damage, immune system disorders, respiratory diseases, and so-called sick building syndrome [5]. Household products and building materials [1,2] are the main indoor sources of formaldehyde. All developed countries have laws to limit the maximum concentration of formaldehyde in building materials. Levels in indoor air are typically in the range of 1–100 ppb (or even higher) depending on formaldehyde source and ventilation [6]. In industry, formaldehyde serves in many processes, e.g., as wood fixative, in paints, insulation foams, dry cleaning solutions, detergents, cosmetics, and pharmaceuticals [7,8]. The World Health Organization (WHO) and the National Institute for Occupational Safety and Health (NIOSH) have set a permissible long-term exposure limit of 0.08- and 1-ppm formaldehyde, respectively. Given the need to monitor formaldehyde concentration in air, portable, cost-effective, robust detectors are highly desirable and have attracted substantial scientific interest, as summarized in a recent review [9]. Many of those sensors are based on inorganic oxidic materials with optimized affinity, but optical approaches have also been published [10]. For introducing shape selectivity, molecularly imprinted polymers (MIPs) [11,12] have attracted increasing attention. In MIP synthesis, the potential analyte acts as a template. Self-assembly of the monomers around this template leads to adapted cavities in the polymer matrix. MIPs have been developed for a wide range of species including small molecules/VOC [13], proteins [14,15,16], and their aggregates [17] up to entire microorganisms [18]. Inherently, there is hardly a size limit for templates. Hence, metal ions are the smallest species for which MIPs have been reported [19,20]. Despite of course being much larger than a metal ion, the formaldehyde molecule is very small compared to other VOC. This is also reflected in the fact that few articles have been published on formaldehyde imprinting so far: The earliest paper on formaldehyde MIPs combined with quartz crystal microbalance (QCM) detection was published in 2005 [21]. It reports appreciable detection limits in a static measuring chamber as well as selectivity factors of about four against larger aldehydes. Another paper reported on pre-concentrating formaldehyde on MIPs for electrophoretic separation [22]. Furthermore, fluoral–polyaniline double layers have been used for formaldehyde sensing [23], even though this approach is not based on molecular imprinting. Two more recent publications in the field of MIP-based formaldehyde sensing rely on more complex materials for recognition: One is based on a MIP-Ag/LaFeO_3_ nanoparticle composite [24], the other one on layer-by-layer assembly of gold nanoparticles and PMAA structures [25]. The former article does not state any selectivity, which makes it difficult to assess the effect of imprinting. The latter indeed reports appreciable imprinting effects. Nonetheless, both systems rely on polymer–nanoparticle composites. Those pre-concentrate formaldehyde on the surfaces of affinity materials, which is an approach that has proven useful also for other volatile pollutants [26]. Herein, we report the design of single-phase recognition materials based on MIPs for assessing formaldehyde vapor concentrations in flow systems for direct gas phase detection of this analyte.

## 2. Materials and Methods

We purchased all chemicals from Sigma-Aldrich and VWR, respectively, in the highest available purity and kept them in storage according to manufacturer recommendations.

### 2.1. MIP Synthesis

**Figure 1:**
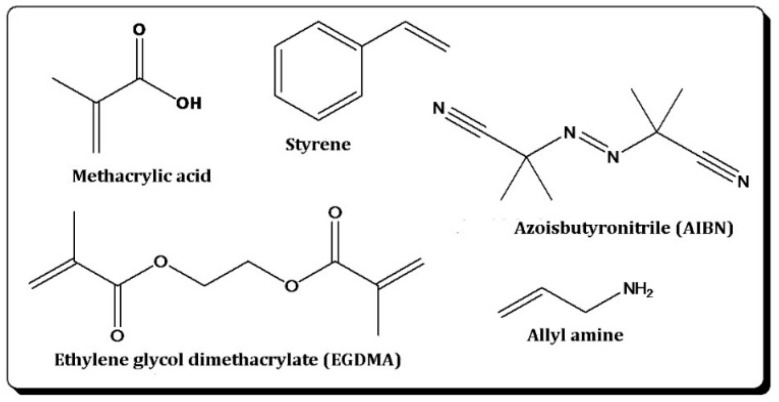
Monomers and reagents used.

The structural formulae of all monomers and reagents are summarized in Figure 1. To synthesize MIPs, we dissolved 13 mg (0.12 mmol) styrene, 13 mg (0.15 mmol) methacrylic acid, and 60 mg (0.31 mmol) ethylene glycol dimethacrylate (EGDMA) in a reaction tube using 500 µL of a binary solvent mixture comprised of 200 µL of methanol and 300 µL of dimethyl formamide (DMF) and added 15 µL of an aqueous formaldehyde solution (37%–38% *w*/*w*, stabilized with 10% methanol). If the polymer contained primary amino groups for recognition (see Section 3.2), the reaction mixture also contained 13 mg (0.26 mmol) allyl amine before adding the template. After obtaining homogeneous, transparent solutions, we added 6 mg of azobisisobutyronitrile (AIBN) as the radical initiator. Polymerization took place by UV irradiation (λ_max_ = 360 nm and 210 W) for 80 minutes until reaching the gel point of the mixture. Non-imprinted polymers (NIPs) were synthesized by the same procedure, replacing the formaldehyde solution with 9 µL of distilled water. Hence, the solvent mixtures of both MIPs and NIPs contain the same amount of water, namely, roughly 2% (9 µL of ~500 µL). For sensor measurements, 15 µL of this oligomer mixture were spin-coated onto one electrode pair of a dual-electrode QCM at 4000 rpm on a custom-made spin-coater, while the second pair was spin-coated with NIPs at the same speed.

For generating MIP NPs, 500 µL of a MIP oligomer solution was pipetted into 10 mL of acetonitrile (AcCN) under vigorous stirring and was then kept on a magnetic stirrer for 12 h. The resulting homogenous NP suspensions were centrifuged at 4000 rpm for ten minutes. The supernatant was removed by pipetting. Then, we re-suspended the particles in 500 µL of AcCN and used this setup to coat devices. For that purpose, 5 µL of the NP suspension was spin-coated at 4000 rpm onto the respective QCM electrode. The spin-coated QCM were kept overnight for drying prior to mass sensitive measurements and atomic force microscopy (AFM) studies. It was not necessary to further cross-link or stabilize the NP layers, because they proved stable during gas-phase measurements.

Surface morphology of NP layers was recorded with a VEECO Instruments Nanoscope IVa in contact mode with silicon nitride tips. NP suspensions were directly spin-coated onto QCM electrodes as described above and assessed after drying overnight.

### 2.2. QCM Manufacturing and Measuring Setup

**Figure 2:**
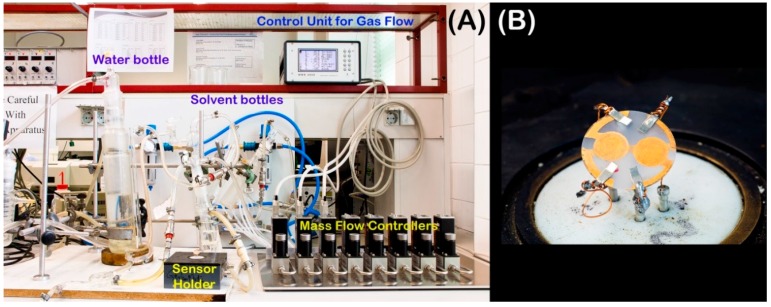
(**A**) Setup used for generating air streams with defined humidity and gas concentration; (**B**) Quartz crystal microbalance (QCM) mounted in measuring cell.

QCM dual electrode structures (f_0_ = 10 MHz) were screen printed with brilliant gold paste containing 12.5% gold colloid (GGP 2093126, Heraeus GmbH, Hanau, Germany) onto AT-cut quartz substrates (13.8 mm in diameter, 168-µm-thick, purchased from Great Microtama Industries, Surabaya, Indonesia). After that, we burned the structures at 400 °C for 4 h to remove organic components and reveal the blank gold electrodes. We then spin-coated the respective recognition material onto an electrode as described above. The QCM was then mounted in a custom-made gas cell (see Figure 2B) and connected to a custom-made oscillating circuit. An Agilent HP5313A frequency counter read out the resonance frequency of the oscillator. Frequency as a function of time was recorded through an Agilent GPIB/USB adapter by a custom-made LabView routine. Gas samples were produced by a gas mixing apparatus consisting of mass flow valves (type RS-485) addressed by a digital controller (Brooks Instruments SLA5850 S, Hatfield, PA, USA). Defined gas streams were bubbled through gas washing flasks containing de-ionized water and analyte, respectively. The entire setup can be seen in Figure 2A.

## 3. Results and Discussion

### 3.1. Development of MIP Thin Films

**Figure 3:**
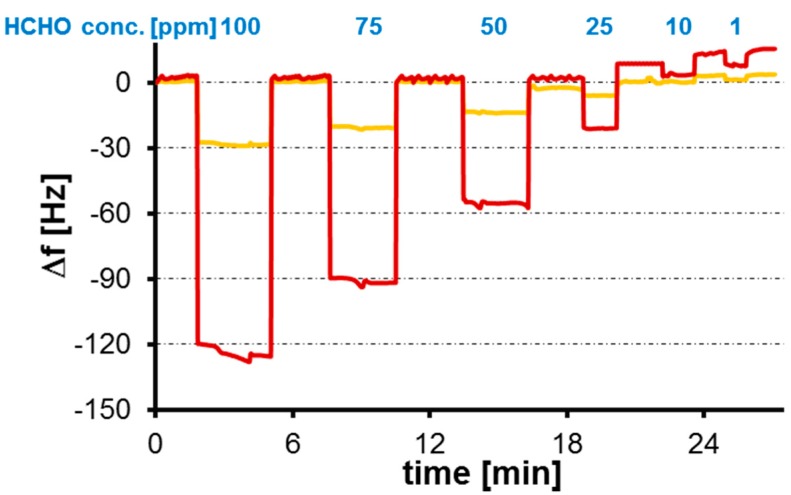
QCM sensor responses of molecularly imprinted polymer (MIP) and non-imprinted polymer (NIP), respectively, to pulses containing different vapor concentrations of formaldehyde.

Previous work has shown that acrylate-based polymers are suitable for formaldehyde MIPs [21]. However, formaldehyde is inherently lipophilic. Hence, our idea was to reduce polarity of the polymer system, but increase polarizability to achieve better interaction between polymer and analyte. For that purpose, we introduced styrene as a monomer. Figure 3 shows the QCM sensor results for a MIP thin film and the respective NIP when exposed to different vapor concentrations of formaldehyde. Evidently, the MIP yield substantially higher frequency shifts at all concentrations than the corresponding NIP. The resulting imprinting effect—i.e., the ratio between MIPs and NIPs—reaches a factor of four, which is appreciably large given the small size of the formaldehyde molecule. Additionally, the magnitude of the frequency shift depends on formaldehyde concentration. Overall, the setup thus leads to a sensor characteristic with a lower limit of detection (LoD) of 1 ppm (S/N ratio ≥ 3). Furthermore, all frequency responses are fully reversible within a few tens of seconds. Especially, in QCM gas phase measurements, the difference in signal between MIPs and NIPs can clearly be attributed to mass effects caused by the incorporation of formaldehyde molecules into recognition sites within the polymer matrix. This again is remarkable given the small size of the formaldehyde molecule.

**Figure 4:**
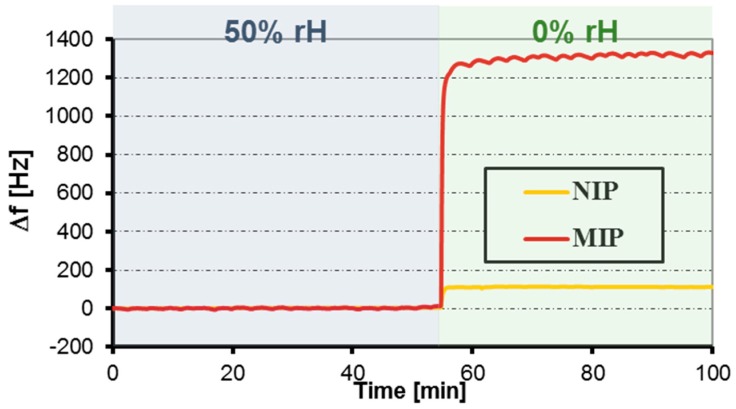
Sensor responses of MIP and NIP, respectively, when switching from an air stream with 50% rH to dry air.

Despite these appreciable results, thin film sensors were not developed further after it turned out that they did not yield useful results in humid air, which makes them useless for operation in real-life conditions. One of the possible reasons of such a response is indicated in Figure 4: switching from 50% rH to dry air changes the frequency of the MIP-coated channel by almost 1400 Hz. It is therefore highly probable that water molecules block access to formaldehyde binding sites within the MIP thin film. Nonetheless, the MIP thin films serve as proof of the principle that appreciable formaldehyde sensor responses can be achieved by this strategy.

### 3.2. Generating MIP Nanoparticles

**Figure 5:**
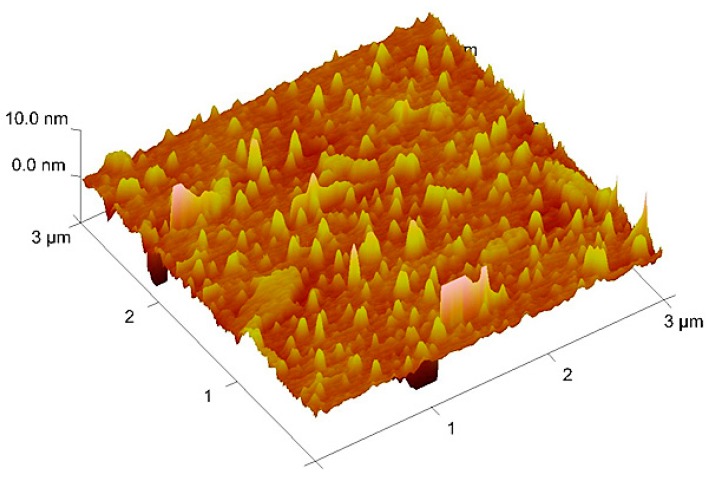
Atomic force microscopy (AFM) image of formaldehyde MIP nanoparticles.

Using MIP NPs seems a rational approach to address these challenges. MIP NPs have drawn increasing attention, because they often yield higher sensitivity and selectivity [27,28] than thin films, partly due to shorter average diffusion pathways. Even at higher humidity, formaldehyde molecules should be able to reach binding sites within the polymer matrix. For that purpose, we synthesized MIP NP by precipitation, because this approach is rather straightforward and does not require additional reagents, such as surfactants. Figure 5 shows an AFM image of a resulting MIP NP layer deposited on a glass substrate.

Evidently, the surface is homogeneously covered with NPs having diameters in the range of 80–150 nm. Precipitation hence was successful and led to the desired particulate material. However, such particles did not yield appreciable responses at elevated humidity. Obviously, the non-specific adsorption of water molecules onto the surface and selective incorporation of formaldehyde molecules compete with one another. Hence, greater affinity between the polymer and formaldehyde is necessary, which can be achieved by modifying the polymer [29]. It is well-known from medical and biological sciences that formaldehyde strongly interacts with primary amino groups (this phenomenon is used for stabilizing tissue etc. post mortem for further investigation) by forming imines (Schiff bases) with primary amino groups of proteins. If the reaction is driven to the end product, this of course leads to a covalent (double) bond between the C of the carbonyl group and the N of the amino group. Hence, we decided to introduce primary amino groups into our material via allyl amine, even though sensing of course aims at reversible interactions. Ab-initio calculations of Hall and Smith [30] revealed that the energy difference between the amine-aldehyde adduct forming in the first step of the reaction and the imine is −46 kJ∙mol^−1^ for methyl amine and formaldehyde in the absence of water. However, the energy of the transition state is +112.3 kJ∙mol^−1^ above the energy of the amine-aldehyde adduct. Such high-energy barrier minimizes the probability of actual imine formation, especially in the gas phase and considering the low amounts of formaldehyde vapors expected in indoor air. Thus, the interactions between formaldehyde and the respective MIP should still be reversible.

**Figure 6:**
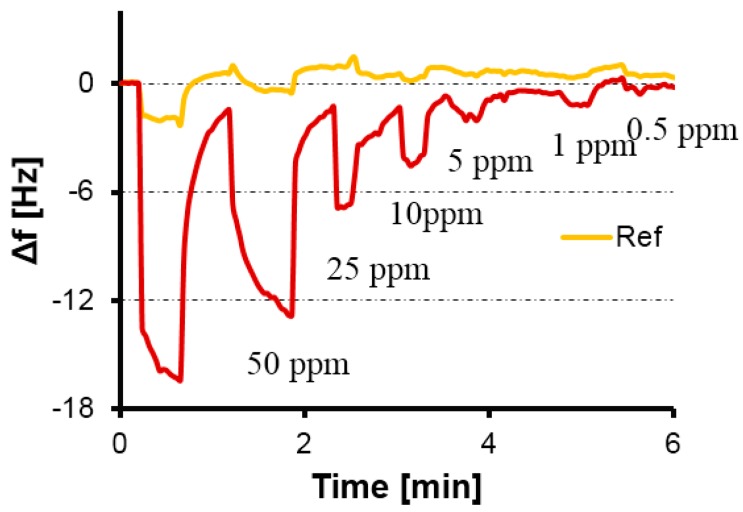
QCM responses of MIP nanoparticles (NPs) containing allyl amine towards formaldehyde at 50% rH.

Adding allyl amine did not change morphology of the nanoparticles: AFM characterization of such NP layers showed particle diameters in the range of around 100–150 nm, which is the same size as for polymers without allyl amine. However, the additional monomer lead to highly appreciable sensor effects, as can be seen in Figure 6. Two aspects are immediately noticeable: Firstly, the material indeed leads to sensitive frequency responses toward formaldehyde vapors despite the fact that the corresponding MIP thin films (data not shown) did not give rise to measureable effects. Secondly, sensors based on MIP NPs respond substantially faster to analyte pulses compared with thin films. According to the data shown in Figure 6, equilibrium is reached within roughly half a minute for each vapor pulse. In the case of MIP thin films (see Figure 3), the same process lasts for around three minutes for each pulse. Again, this strongly suggests that accessibility of the respective binding sites in the material plays a seminal role in sensing. In the case of nanoparticles with a roughly 100-nm diameter, possible diffusion pathways are in the range of at least several tens of nanometers. Thin films, however, are typically 100–200 nm high on each electrode (as determined by network analyzer measurements) and have a diameter of roughly 6 mm (electrode diameter after screen printing is 5 mm, and the layers are somewhat wider). Thus, both the accessible surface is much lower than for NP, and diffusion pathways within the polymer can be expected to be much longer. The MIP NP sensor reaches LoD = 0.5 ppm formaldehyde vapor in humid air, which is the same as that for the thin film at dry conditions. During all our measurements (same quartz over several days), sensor signals returned to their respective baselines, strongly indicating reversible—i.e., non-covalent—binding.

### 3.3. Selectivity

Of course, sensitivity alone is not sufficient to claim successful imprinting. For that, selectivity must also be assessed. Table 1 summarizes the outcome of these experiments for both thin films and nanoparticle layers toward 100-ppm pulses of a range of compounds each.

**Figure 7:**
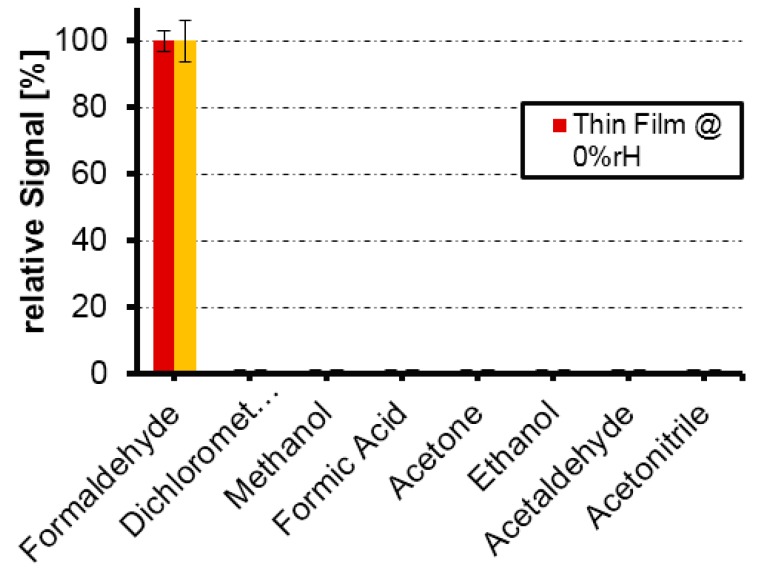
Selectivity patterns of MIP thin films and MIP NP layers showing relative signals.

All those compounds are either somewhat similar in size or are chemically related to formaldehyde: Methanol and formic acid are the reduction and oxidation product of formaldehyde, respectively. Acetaldehyde also contains the CHO group and acetone at least the carbonyl functionality. The dichloromethane molecule is just slightly larger than formaldehyde. Surprisingly, none of these compounds gave rise to any sensor response revealing specific behavior of the sensor, as all frequency shifts stayed below the noise level of 1 Hz on the oscillator. For better visualization, Figure 7 summarizes the normalized sensor responses for these two systems: the frequency shifts in this case are related to the sensor response toward the 100-ppm formaldehyde. Error bars for formaldehyde responses correspond to standard deviations obtained for three parallel measurements. In the case of frequency shifts that are not statistically significant, the noise level of the oscillator was used to determine the errors.

As can be seen, selectivity is the same in both cases, i.e., for thin films containing no allyl amine as well as nanoparticles containing allyl amine. The data also represents results obtained at two different humidity levels. Although of course not directly comparable, one can clearly see that the sensor surprisingly reacted specifically towards formaldehyde: no statistically significant signal could be observed for any other VOC except formaldehyde during triplicate measurements of those compounds. The comparably small size of the formaldehyde molecule combined with its very distinct functionality may be the reason for such unusually high selectivity, which we previously only observed once in non-covalent MIPs [31]. Both sensitivity and selectivity results very strongly corroborate the outstanding abilities of the imprinting procedure: the MIPs can distinguish between molecules that are rather similar in size, such as formaldehyde and methanol. They also discriminate according to size: acetaldehyde hardly yields any effects on the MIP layer despite containing the same functional group as formaldehyde.

## 4. Conclusions

We herein present a gas sensor for formaldehyde detection based on MIP, relying on both thin films and nanoparticles, respectively. It can detect vapor concentrations down to 500 ppb with outstandingly high selectivity. Optimal results are obtained with MIP NP. The latter point especially reaches beyond immediate science: MIP NP can be introduced into production processes of sensors fairly straightforwardly and thus, in principle, open up a way for commercializing such systems.

## Figures and Tables

**Table 1:** Sensor responses of MIP thin films and NPs (containing allyl amine in the monomer mixture) at 0% rH and 50% rH, respectively, toward 100-ppm formaldehyde (HCHO), dichloromethane (CH2Cl2), methanol (MeOH), formic acid, acetone ethanol (EtOH), acetaldehyde (AcCHO), and acetonitrile (AcCN).

Sensor	HCHO	CH_2_Cl_2_	MeOH	Formic Acid	Acetone	EtOH	AcCHO	AcCN
Thin film (0% rH)	−65 ± 2 Hz	Below noise	Below noise	Below noise	Below noise	Below noise	Below noise	Below noise
NP (50% rH)	−24 ± 1 Hz	Below noise	Below noise	Below noise	Below noise	Below noise	Below noise	Below noise

## Abbreviations

The following abbreviations are used in this manuscript:

AcCN	acetonitrile
AFM	atomic force microscopy
AIBN	azoisobutyronitrile
DMF	dimethyl formamide
EGDMA	ethylene glycol dimethacrylate
MIP	molecularly imprinted polymer
NIP	non-imprinted polymer
NP	nanoparticles
QCM	quartz crystal microbalance
rH	relative humidity
VOC	volatile organic compound
